# Ready-to-Use Therapeutic Foods (RUTFs) Based on Local Recipes Are as Efficacious and Have a Higher Acceptability than a Standard Peanut-Based RUTF: A Randomized Controlled Trial in Indonesia

**DOI:** 10.3390/nu15143166

**Published:** 2023-07-17

**Authors:** Asrinisa Rachmadewi, Damayanti D. Soekarjo, Blandina Rosalina Bait, Julia Suryantan, Rivani Noor, Jee Hyun Rah, Frank T. Wieringa

**Affiliations:** 1 Savica Consultancy, Kota Jakarta Selatan 12440, Indonesia; 2 UNICEF Indonesia WTC 2, Jakarta 12920, Indonesia; 3 Directorate of Nutrition and Maternal and Child Health, Ministry of Health, Jakarta 12950, Indonesia; 4 UMR QualiSud, Institut de Recherche pour le Développement (IRD), 34394 Montpellier, France; 5 Qualisud, University of Montpellier, Avignon University, CIRAD, Institut Agro, IRD, Université de la Réunion, 34394 Montpellier, France

**Keywords:** malnutrition, ready-to-use therapeutic foods, SAM, RUTF, Indonesia, local production, children, acceptability

## Abstract

To strengthen community-based treatment of severe acute malnutrition (SAM) in Indonesia, locally produced ready-to-use therapeutic foods (RUTFs) are needed, but data on their acceptability and effectiveness are lacking. We conducted an individually randomized controlled trial in 302 children (6–59 months old) with uncomplicated SAM receiving 8 weeks of a standard RUTF (CON) or one of four alternative RUTFs produced with locally available ingredients: soybean (SOY), mungbean (MUN1, MUN2) or peanuts (PEA). The main outcomes were weight gain and product acceptability. Children consumed on average 2.2 kg of standard RUTF, but up to 4.5 kg of the local products (MUN2, *p* < 0.05). Mean weight gain did not differ across the groups (*p* > 0.05). Controlled for consumption, children receiving either CON or SOY RUTF gained >2 g/kg body weight (BW)/day compared with 1.6 g/kg BW/day in children receiving the other RUTF products (*p* > 0.05). Overall drop-out was 29.1%, ranging from 21.3% (MUN2) to 38.3% (CON, *p* > 0.05). Mean time to drop out was 19 days in the CON group, significantly shorter than in the PEA group (33.6 days, *p* < 0.05). Thus, with no difference in weight gain and better acceptance, the development of locally produced RUTFs in Indonesia is warranted to strengthen the community-based treatment of SAM.

## 1. Introduction

Indonesia has an estimated overall caseload of over 6 million children under 5 years of age with wasting, of whom more than 2 million are severely wasted, based on national basic health research data [1,2]. Severe acute malnutrition (SAM) is defined by a weight-for-height Z-score (WHZ) <−3 and/or a mid-upper arm circumference (MUAC) <115 mm, and/or nutritional oedema, regardless of anthropometric status [3]. Children with severe wasting are up to 12 times more likely to die compared with their well-nourished peers and are susceptible to long-term negative health outcomes such as linear growth impairment, leading to stunting and sub-optimal cognitive development [4]. Therefore, children diagnosed with SAM require immediate treatment. 

The Government of Indonesia has shown great commitment to fighting wasting through a rapid expansion of integrated management of acute malnutrition (IMAM) services to prevent and treat wasting in Indonesia, including strengthening inpatient- and community-based treatments. Inpatient treatment of severe wasting has been a standard component of health services in Indonesia for many years. However, in the >85% of cases that have no complications, SAM can also be treated at home using high-energy-dense ready-to-use therapeutic foods (RUTFs). Indeed, research has shown that home treatment through IMAM is at least as effective as in-patient treatment for uncomplicated SAM [5,6]. While the effectiveness of RUTFs on weight gain and recovery rates has been well established, reported weight gains are often less than the 4 g/kg body weight (BW)/day required by WHO [7]. Besides providing basic medicines such as antibiotics and deworming tablets, RUTFs are an essential part of IMAM [4]. However, locally produced RUTFs are currently not yet available in Indonesia and the importation of RUTFs is restricted, posing major challenges to the implementation of IMAM at scale. 

RUTF is defined as a high-energy, fortified, ready-to-eat, soft or crushable, non-water-based food suitable for the treatment of children with SAM from the age of six months onward [8]. The original WHO guidelines required at least 50% of its protein content to come from dairy [3], although this requirement has recently been adapted [9]. The most widely used RUTF is a peanut paste with milk and oil as well as vitamins and minerals, containing approximately 540 kcal/100 g. The peanuts may be replaced with other legumes or cereals, depending on local availability, cost, and acceptability, while maintaining compliance with the recommended nutritional composition [3]; however, the introduction of local RUTFs often requires a long development process [10]. 

To support the efforts of the Government of Indonesia in scaling up IMAM to the national level, UNICEF, Savica and the French National Research Institute for Sustainable Development (IRD) conducted a combined acceptability and efficacy study on different types of RUTFs produced using local ingredients that are readily available in Indonesia. Here we report the main outcomes of the study, including acceptability and changes in weights and MUACs of children treated for SAM using standard RUTF or one of four local RUTFs.

## 2. Materials and Methods

### 2.1. Study Design

This study was designed as an individually randomized controlled trial. All children meeting the inclusion criteria were invited to participate in the intervention study. Each child was randomly allocated to receive 8 weeks of SAM treatment with one of five RUTF products. A peanut–milk paste RUTF (CON) was used as the control RUTF. Experimental RUTFs consisted of a soy–milk paste (SOY), a mungbean–milk paste (MUN1), a slightly thinner mungbean–milk paste (MUN2) or a peanut–milk paste-filled wafer roll (PEA). Details of the nutritional content of the products are provided in Table 1. Products conforming to WHO guidelines and with certificates of analysis were submitted by all producers. According to protocol, randomization was conducted based on an automatically generated allocation list with a block size of ten; however, due to the late production of one product, the first two weeks of the trial started without this product (block randomization with eight blocks), and the product was slightly overrepresented in the later blocks to compensate.

### 2.2. Study Location

The study was conducted in Bogor District, West Java Province, and covered 322 villages and 81 Primary Healthcare Centres (PHC) in 31 subdistricts. This represents around 80% of this district. The remainder of the subdistricts were too remote and/or too difficult to access. The study was conducted from July to December 2021.

### 2.3. Subjects

Eligible for inclusion in the study were children aged 6–59 months with uncomplicated SAM (WHZ < −3 and/or MUAC < 115 mm and/or presence of bilateral pitting oedema +1 or +2 regardless of anthropometry) who had no underlying health conditions or complications of SAM, no severe anaemia (<70 g/L) or body weight less than 4 kg, who were not allergic to any of the ingredients in the RUTFs, who passed the appetite test and who had not received treatment for SAM (including not having consumed RUTF, F75, F100 or food for special medical purposes) in the last two months.

**Figure 1:**
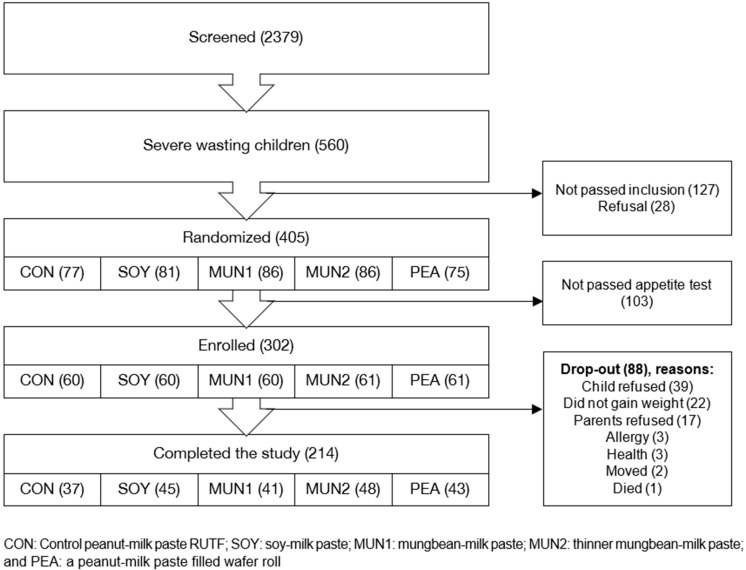
Study flowchart.

As reliable data from the PHC on the anthropometric status of children in their catchment area were not available due to the COVID-19 pandemic (no monthly growth monitoring sessions being conducted), we pre-screened children for SAM using MUAC through home visits, using a cut-off of 135 mm, to allow better detection of children with low WHZ [11]. This approach generated a short list of children at high risk of SAM. Children identified during the pre-screening were then re-visited at home, in close coordination with the PHC staff and with full adherence to national and local regulations and protocols related to the COVID-19 pandemic. The screening process (Figure 1) consisted of confirming SAM based on anthropometry (WHZ and/or MUAC and/or nutritional oedema), physical examination, determination of haemoglobin concentration (HemoCue™ Angelholm, Sweden 201+) and an appetite test. Eligible children whose parents gave informed consent were enrolled in the study.

### 2.4. Study Procedures and Data Collection

#### 2.4.1. Anthropometry

At recruitment, height or length was measured to the nearest 0.1 cm using a Shorrboard^®^ (Weigh and Measures, LLC, Olney, MD, USA). Weight was measured to the nearest 0.1 kg (SECA 874 or AND UC-321 digital weighing scales). MUAC was measured to the nearest 1 mm using standardized measuring tapes (UNICEF). All measurements were performed in duplicate and repeated if they exceeded the allowable difference (>0.2 cm for height/length, >0.2 kg for weight and >2 mm for MUAC). Weight and MUAC measurements were repeated weekly, whereas heights/lengths were measured at 4 weeks (mid-term) and 8 weeks (end of study). All children were screened for oedema using standardized techniques.

#### 2.4.2. Appetite Test

Prior to the appetite test, eligible children were randomized to one of the five intervention arms using the block randomization described above. The criteria to pass the appetite test were based on the Ministry of Health (MoH) Guidelines on SAM treatment. Each child was given one sachet of their assigned product by the mother/caregiver, and consumption was observed for up to 30 minutes, after which leftovers were weighed. Duration of consumption, the total amount of product consumed, the observed reaction of the child and information on the child’s normal appetite were recorded. If a child failed the appetite test and generally had very poor appetite, the child was excluded and referred to the PHC for follow-up and treatment. However, if the mother stated that her child usually had good appetite, they were provided with the product to consume at home for three days. If the child still did not consume the RUTF (or consumed <50% of the provided RUTF for three consecutive days), the child was dropped from the study and referred to the PHC for treatment.

#### 2.4.3. Data Collection

At baseline, a short questionnaire on the social-economic status of the household, household food security, infant and young child feeding (IYCF) practices (including 24-h food recall), use of micronutrient supplements and supplementary feeding provided through the PHC, morbidity (history and current), gestational age, birth weight and length and immunization status was administered. All data were recorded on tablets using a digital questionnaire (Survey2 Go). At the endline, data on 24-h food recall, height, weight, MUAC, haemoglobin concentration and parents’ observation of their child’s preferences (taste, colour and smell) of the RUTF products were collected.

Mothers/caregivers were provided with a monitoring book—and an explanation of its use—to record the frequency and timing of RUTF consumption, frequency of breastfeeding and intake of any other foods on a daily basis. A field worker made weekly home visits to collect these recordings, as well as information on intra-household sharing of the RUTF and morbidity (weekly recall). The field worker counted the remaining sachets and weighed any open sachets to calculate the total amount of RUTF product consumed. During this visit, the field worker also provided counselling to the mothers/caregivers on RUTF consumption, including not sharing the RUTF with other household members and giving other foods only after the child had finished their daily dose of RUTF. In addition, field workers maintained regular communication with the mothers/caregivers through texting and phone calls. Phone credit was provided to mothers/caregivers and cadres for this purpose. 

They reminded mothers/caregivers to feed their child the RUTF, fill in the monitoring book and addressed any problems that arose. On the day after the field worker’s visit, mothers/caregivers who were able to do so were asked to take a picture of the package and their entry in the monitoring book so that the field workers could provide remote feedback. Mothers/caregivers without a camera phone were visited three times in the first week to make sure they completed the monitoring book correctly.

#### 2.4.4. Treatment

A weekly supply of RUTF was provided in a box during each of the weekly check-up visits, adjusted each week to the weight of the child according to the MoH Guidelines on SAM treatment (Table 2).

Each sachet was kept in a zip-lock plastic bag labelled with the name of the child, the name of the mother/caregiver, week, day and date of consumption. Mothers/caregivers were given counselling on RUTF consumption. In addition, the child also received broad-spectrum antibiotics (amoxicillin) for five days in the first week of the intervention and anthelminthic therapy (pyrantel palmoate) according to the MoH Guidelines on the treatment of severe wasting, except for those who had received deworming in the past six months.

### 2.5. Sample Size Calculation

The main objective of the study was to compare four locally produced RUTFs with the standard peanut-based RUTF for acceptability and efficacy. For the efficacy study, we based our initial sample size estimates on an overall increase of 4 g/kg BW/day, the target set by the WHO. A sample size of 50 children per group would allow the detection of a 20% difference between control and intervention products, assuming a standard deviation (SD) of 1.6 g/kg BW, a study design effect of 1.0 (individual randomization), a significance of 0.05 and a power of 0.80.

### 2.6. Statistical Analyses

All data were entered into a digital questionnaire and extracted to SPSS 25 and cleaned. Data from 302 enrolled children were included in the analysis. Descriptive analyses were employed to examine the distribution of the full range of variables. All enrolled children were included in the ‘intention to treat’ analysis, while a separate analysis was conducted on the children who finished the 8-week intervention study. Repeated-measurement data were analysed using general linear models (GLM). Normality of data was checked using z-score skewness and kurtosis, and the Kolmogorov-Smirnov test. Data are presented as the mean ± standard deviation (SD) for normally distributed variables, median and interquartile range (IQR) for non-normally distributed variables and as percentages for categorical variables. Non-parametric Kruskal–Wallis test was used to test differences between non-normally distributed variables. Pearson chi-square test was used to compare categorical variables. Mann–Whitney U test was used to compare non-parametric variables between intervention groups. Data were considered significant at *p* < 0.05.

Minimum dietary diversity score (MDDS) and minimum acceptable diet (MAD) were calculated using WHO IYCF Practices Indicators 2010 and 2021. A dichotomous variable was created to define whether children met the MDDS (at least 4 food groups) or not (<4 food groups). Household food security was determined by applying the Household Food Insecurity Access Scale.

### 2.7. Quality Control

All field staff held a degree in nutrition, with experience in conducting research and taking anthropometric measurements in young children. Prior to the fieldwork, the field team was given standardized training on the study methodology, IMAM and counselling on RUTF consumption. Data were collected using a digital questionnaire, with multiple quality checks throughout the process. All equipment was cleaned and calibrated regularly using a standardized procedure. Any malfunctioning equipment was replaced with a spare. Dropouts and Adverse Events/Serious Adverse Events (AE/SAE) were recorded and reported to UNICEF and the health authorities.

## 3. Results

A total of 6145 children were pre-screened and 2379 were identified as being at risk of SAM. Of these, 560 children had SAM; a quarter did not meet the study criteria (*n* = 127; 22%) and were referred to the PHC for treatment or their parents did not consent (*n* = 28; 5%) (Figure 1).

Among the 302 (74.6%) enrolled children, 253 (83.8%) had WHZ scores < −3, 18 (6.0%) had MUAC < 11.5 cm, and 31 (10.3%) met both inclusion criteria (Table 3). No child had bilateral pitting oedema. There were no differences in the enrolment or the drop-out rates between the children who were enrolled based on their WHZ score, MUAC or both.

Overall drop-out rate during the intervention was high at 29.1% (*n* = 88), ranging from 21.3% in the MUN2 group to 38.3% in the group receiving the control RUTF (CON). While this was not statistically significant (*p* = 0.30), children in the CON group dropped out significantly earlier compared with children in the PEA group (19.0 vs. 33.6 days; *p* = 0.002, GLM, Table 4). Additionally, children in the MUN2 group stayed in the study longer compared with children in the CON group (24.3 days, *p* = 0.030, Log Rank); similar trends were observed for children in the SOY group (*p* = 0.078) and the PEA group (*p* = 0.143).

There was no preference among the caregivers for the taste of the products (*p* > 0.05), although slightly more caregivers of children who received the MUN2 and PEA products indicated that their children liked the taste compared with the other products. 

The amount of RUTF consumed during the appetite test (20.0 ± 14.9 g) and the velocity of RUTF consumption (0.75 g/min) were not different among the 5 intervention groups (*p* > 0.05). However, intake was significantly different over the 2 months of SAM treatment, with children receiving MUN2 consuming twice as much as children receiving the CON or SOY RUTF (4.49 kg vs. 2.18 kg and 2.32 kg, respectively, *p* < 0.05, Table 5).

Mothers were encouraged to continue breastfeeding their children during the intervention. Only 7% of the children consumed other foods less than once per day during the 8-week intervention, while the rest of the children consumed other foods 1–3 times per day, despite not finishing their daily dose of RUTF.

The mean weight gain, expressed as g/kg BW/day, ranged from 1.38 ± 0.19 g in the PEA group to 1.67 ± 0.19 g in the MUN2 group over the 8-week intervention (*p* > 0.05). Average consumption over the course of the study was significantly different between the groups. When controlled for consumption, age and food insecurity, the estimated increase in the weight of children receiving either CON or SOY RUTFs was around 2.3 g/kg BW/day compared with around 1.6 g/kg BW/day in children receiving the other RUTF products (*p* > 0.05). 

Weight gain (g/kg BW/day) was not statistically different (*p* = 0.8) between children admitted with low MUAC only (1.61 g/kg BW/day), low WHZ only (1.45 g/kg BW/day) or both low MUAC and low WHZ (1.58 g/kg BW/day). Additionally, the recovery of children included in the study based on MUAC only or WHZ only did not differ (67% and 76% recovered, respectively, *p* > 0.05).

At baseline, the mean haemoglobin concentration in the study population was 9.9 ± 1.3 g/dL (*n* = 296 children) and there were no significant differences between the groups. A slight increase in haemoglobin concentration between baseline and endline was seen in all groups (*n* = 210), but the increase was significantly higher in the MUN2 group compared with the PEA and MUN1 RUTF groups.

## 4. Discussion

To our knowledge, this study is the first large trial in Indonesia comparing the acceptability and effectiveness of RUTFs based on locally available ingredients compared with a standard RUTF. This study showed that RUTF products made from locally available ingredients were comparable to the standard peanut-based RUTF in terms of weight gain. Weight gain was between 1.3–1.6 g/kg BW/day, which is in line with findings of effectiveness trials in Cambodia and Vietnam [12,13,14], but much less than the 4.0 g/kg BW/day set by the WHO. One factor affecting this lower-than-required weight gain was compliance, especially in the standard and soy-based RUTFs. The low compliance is worrisome, as caregivers and children received intensive guidance from field workers and supervision was much higher than can be expected in a standard IMAM program. We assume that weight gain would have been higher if compliance had been higher, but in real program settings, lower compliance is more likely to be encountered, yet, all products met the WHO cure rate criteria of 70%. An important finding of the present study, therefore, is that compliance was significantly better for some of the local products, clearly showing the importance of developing RUTF products adapted to local taste preferences.

More than 60% of the children in this study were less than 2 years old, indicating that severe wasting is a serious public health problem during early childhood in Indonesia. Recent evidence from Africa and South-East Asia shows that children who experience a period of wasting are at an increased risk of stunting and mortality in the period thereafter. Hence, given that the majority of severe wasting occurs within the window of opportunity to reduce stunting, early detection and treatment of severe wasting will contribute significantly to the efforts of the Government of Indonesia to reduce stunting. The importance of counselling, both on IYCF and continued severe wasting treatment, cannot be underestimated, with most dropouts in the <2 years age group and >40% of children in the control group not finishing treatment.

Most products seem to be acceptable, in particular, local products and especially the MUN2 and PEA products were well received by the younger children (6–11 months old); the PEA RUTF was crushed by the caregivers before presentation to their children when the child was below 11 months of age. The MUN2 product, which had lower viscosity than the other pastes, was less well received by older children (24–59 months). The relatively lower acceptability of CON RUTF is in line with findings in other South-East Asian countries [10,12,13].

Based on the findings of this study, the successful implementation of an IMAM program in Indonesia will depend on the production and distribution of local RUTF products, because the almost 40% drop-out within the first weeks of SAM treatment observed with the current gold standard (peanut–milk paste RUTF) is untenable. Therefore, local development and production of RUTF should be strongly encouraged. Further revision of the products in this study is recommended to further increase the nutrient density and acceptability of the local products. The availability of a larger choice of products can increase the success of a larger national program as it enables catering to personal and local preferences in terms of ingredients, taste and form, and can be adapted to the age of the child.

The lack of impact for 4 of the 5 RUTFs is in line with studies from Africa and Asia, which showed little or no improvement in haemoglobin concentrations in children receiving SAM treatment with standard RUTF [15,16] containing 10–14 mg iron/100 g. Some researchers have argued that the amount of iron in standard RUTF should be increased to over 30 mg/100 g [17] to see an impact on iron status in children recovering from SAM, but concerns about the negative effects of high doses of iron on e.g., human microbiome, might hamper this approach.

It is important to note that close monitoring and intensive counselling are essential for the success of SAM treatment at home. Not only should the importance of the treatment be stressed, but counselling should include infant and young child feeding, given the high proportion of children under 2 years who have SAM. Therefore, government programs should consider incorporating Social and Behavioural Change Communication (SBCC) and a ‘buddy’ system similar to that used in tuberculosis programs to motivate the parents and ensure optimal compliance.

Overall, the results of the present study confirm that the development of RUTFs adapted to the local availability of ingredients and local organoleptic preferences contribute to higher acceptance of the products and thereby can contribute to more efficient IMAM programs.

## Figures and Tables

**Table 1:** Nutritional content of the RUTF products.

Nutritional Content per 100 g	Products				
	CON	SOY	MUN1	MUN2	PEA
Energy (kcal)	543	543	543	523	534
Proteins (g)	13.1	14.0	13.7	12.2	13.5
Fat (g)	32.3	35.7	34.7	29.1	31.6
Carbohydrates (g)	No information	No information	44.1	52.7	49.1
Iron (mg)	13.0	10.0	13.5	15.6	6.1
Vitamin C (mg)	119.9	118.1	109.9	55.6	5.2

CON: Control peanut–milk paste RUTF; SOY: soy–milk paste; MUN1: thick mungbean–milk paste; MUN2: thinner mungbean–milk paste; PEA: a peanut–milk paste-filled wafer roll.

**Table 2:** RUTF rations based on body weight.

Weight (kg)	Sachets/Day	Sachets/Week	kcal/Day
4.0–4.9	1.5	10	750
5.0–6.9	2	15	1000
7.0–9.9	3	20	1500
10.0–14.9	4	30	2000

**Table 3:** Characteristics of the study population at baseline ^1^.

Characteristics	CON	SOY	MUN1	MUN2	PEA	Total
	*n* = 60	*n* = 60	*n* = 60	*n* = 61	*n* = 61	*n* = 302
Sex, male (*n* (%))	35 (58.3)	34 (56.7)	30 (50.0)	32 (52.5)	37 (60.7)	168 (55.6)
Age (months)	22.8 ± 12.2	25.8 ± 13.9	21.3 ± 12.0	22.6 ± 12.3	23.2 ± 12.6	23.2 ± 12.6
6–11 (*n* (%))	9 (15.0)	7 (11.7)	13 (21.7)	9 (14.8)	9 (14.8)	47 (15.6)
12–23 (*n* (%))	30 (50.0)	23 (38.3)	28 (46.7)	31 (50.8)	26 (42.6)	138 (45.7)
24–59 (*n* (%))	21 (35.0)	30 (50.0)	19 (31.7)	21 (34.4)	26 (42.6)	117 (38.7)
Height (cm)	76.2 [9.5]	76.1 [11.8]	73.7 [10.0]	76.3 [11.7]	76.1 [11.8]	76.2 [10.9]
Weight (kg)	7.4 [1.5]	7.2 [1.8]	6.9 [1.7]	7.1 [2.0]	7.2 [1.8]	7.3 [1.7]
MUAC (cm)	12.5 [1.2]	12.4 [1.2]	12.1 [1.1]	12.2 [1.1]	12.4 [1.2]	12.3 [1.2]
WHZ	−3.28 [0.40]	−3.25 [0.52]	−3.30 [0.42]	−3.32 [0.52]	−3.25 [0.52]	−3.29 [0.48]
Hb (g/dL)	10.06 ± 1.30	9.97 ± 1.39	10.01 ± 1.17	9.90 ± 1.25	9.67 ± 1.12	9.92 ± 1.25
Gestational age (week)	37.7 ± 3.0	37.8 ± 3.9	37.8 ± 3.0	38.0 ± 2.7	37.7 ± 4.1	37.8 ± 3.4
Birth weight (kg)	2.71 ± 0.59	2.83 ± 0.34	2.70 ± 0.60	2.72 ± 0.51	2.70 ± 0.63	2.73 ± 0.54
Birth length (cm)	47.7 ± 2.7	47.4 ± 2.3	46.7 ± 3.4	47.3 ± 2.9	47.5 ± 2.2	47.3 ± 2.7
Morbidity history 3 days (*n* (%))						
Fever	6 (10.0)	5 (8.3)	3 (5.0)	2 (3.3)	3 (4.9)	19 (6.3)
Cold (*p* = 0.002)	19 (31.7)	11 (18.3)	10 (16.7)	21 (34.4)	5 (8.2)	66 (21.9)
Diarrhoea	2 (3.3)	2 (3.3)	2 (3.3)	3 (4.9)	2 (3.3)	11 (3.6)
Vomit	0 (0.0)	5 (8.3)	3 (5.0)	2 (3.3)	2 (3.3)	12 (4.0)
Rash	4 (6.7)	6 (10.0)	2 (3.3)	1 (1.6)	3 (4.9)	16 (5.3)
Constipation	1 (1.7)	1 (1.7)	0 (0.0)	0 (0.0)	0 (0.0)	2 (0.7)
Household						
Household Food Insecurity (*n* (%))	37 (61.7)	47 (61.7)	43 (71.7)	41 (67.2)	45 (73.8)	203 (67.2)
Severely food insecure (*n* (%))	15 (25.0)	10 (16.7)	9 (15.0)	17 (27.9)	19 (31.1)	70 (23.3)
Mother main caregiver (*n* (%))	60 (100.0)	60 (100.0)	59 (98.3)	58 (95.1)	58 (95.1)	295 (97.7)
Maternal age, years	30.0 [12]	30.0 [10]	30.0 [11]	32.0 [7]	30.0 [10]	31.0 [9]
Maternal education, years	9.0 [6]	9.0 [6]	7.0 [6]	9.0 [6]	9.0 [6]	9.0 {6]
Paternal age, years	35.0 [11]	34.5 [9]	35.0 [11]	37.0 [8]	34.5 [9]	36.0 [10]
Paternal education, years	9.0 [6]	9.0 [6]	6.0 [6]	9.0 [6]	9.0 [6]	9.0 [6]
Feeding and caring practices						
Ever breastfed (*n* (%))	56 (93.3)	59 (98.3)	58 (96.7)	60 (98.4)	59 (96.7)	292 (96.7)
Early initiation of breastfeeding < 1 h (*n* (%))	34 (60.7)	29 (49.2)	32 (55.1)	40 (66.6)	25 (52.6)	126 (56.9)
Currently breastfed (*n* (%)) (*p* < 0.05)	36 (64.3) ab	25 (42.4) a	41 (70.7) b	36 (60.0) ab	34 (57.6) ab	172 (58.9)
Complementary foods first introduced (*n* (%))						
<3 months	32 (53.3)	43 (71.1)	32 (53.3)	34 (55.7)	42 (68.9)	183 (60.6)
4–6 months	14 (23.3)	8 (13.3)	8 (13.3)	10 (16.4)	8 (13.1)	48 (15.9)
Prelacteal feeds given in first 2 days of life (*n* (%))	28 (46.7)	31 (61.7)	31 (51.7)	32 (52.5)	40 (65.6)	168 (55.6)
Fully immunized according to age (*n* (%))	3 (5.0)	3 (5.0)	3 (5.0)	0 (0.0)	1 (1.6)	10 (3.3)
Received Vitamin A in the past 6 months (*n* (%))	38 (82.6)	33 (82.5)	34 (79.1)	30 (81.1)	38 (88.4)	173 (82.8)
Received deworming in the past 6 months (*n* (%))	16 (29.1)	12 (20.7)	10 (19.2)	6 (12.2)	6 (11.3)	50 (18.7)

^1^ Data are presented as mean ± SD for normally distributed variables, as median [IQR] for non-normally distributed variables and as *n* (%) for categorical variables; all *p*-values > 0.05, unless mentioned; differences between groups are indicated by different letters (the group indicated with ‘a’ is significantly different (*p* < 0.05) from the group indicated with ‘b’, but not from the group indicated with ‘ab’). IQR: Interquartile Range; CON: Control peanut–milk paste RUTF; SOY: soy–milk paste; MUN1: thick mungbean–milk paste; MUN2: thinner mungbean–milk paste; PEA: a peanut–milk paste-filled wafer roll.

**Table 4:** Number of children (% of total) and days until drop-out before end of treatment (56 days).

Days	Product				
	CON	SOY	MUN1	MUN2	PEA
*n* (% of total)	23 (38.3%)	15 (25%)	19 (31.7%)	13 (21.3%)	18 (29.5%)
Mean stay until drop-out (day)	19.0	27.5	21.4	24.3	33.6
Minimum	6	7	3	7	14
Maximum	42	49	49	42	49

CON: Control peanut–milk paste RUTF; SOY: soy–milk paste; MUN1: thick mungbean–milk paste; MUN2: thinner mungbean–milk paste; PEA: a peanut–milk paste-filled wafer roll.

**Table 5:** RUTF consumption and caloric and protein intake over the 8-week intervention (*n* = 214).

	CON	SOY	MUN1	MUN2	PEA
*n*	37	45	41	48	43
Mean RUTF consumption (kg)	2.18 (±2.02) ^a^	2.32 (±2.35) ^a^	2.90 (±2.49) ^ab^	4.49 (±3.57) ^b^	3.85 (±2.63) ^ab^
Mean caloric intake from RUTF per day (kcal)	211.58 ± 196.05	225.16 ± 228.08	281.46 ± 241.66	419.09 ± 333.22	367.47 ± 251.02
Mean protein intake from RUTF per day (g)	5.10 ± 10.30	5.80 ± 13.63	7.09 ± 17.67	9.78 ± 34.92	9.28 ± 24.41

Differences between groups are indicated by different letters (the group indicated with ‘a’ is significantly different (*p* < 0.05) from the group indicated with ‘b’, but not from the group indicated with ‘ab’). CON: Control peanut–milk paste RUTF; SOY: soy–milk paste; MUN1: thick mungbean–milk paste; MUN2: thinner mungbean–milk paste; PEA: peanut–milk paste-filled wafer roll.

## Data Availability

The data that support the findings of this study are available from UNICEF Indonesia, but restrictions apply to the availability of the data, which were used under license for the current study, and therefore are not publicly available. Data are available from the authors upon reasonable request and with permission from UNICEF Indonesia.
